# The Hippo component YAP localizes in the nucleus of human papilloma virus positive oropharyngeal squamous cell carcinoma

**DOI:** 10.1186/s40463-017-0187-1

**Published:** 2017-02-22

**Authors:** Faisal Alzahrani, Leanne Clattenburg, Shanmugam Muruganandan, Martin Bullock, Kaitlyn MacIsaac, Michael Wigerius, Blair A. Williams, M. Elise R. Graham, Matthew H. Rigby, Jonathan R. B. Trites, S. Mark Taylor, Christopher J. Sinal, James P. Fawcett, Robert D. Hart

**Affiliations:** 1 Division of Otolaryngology, Department of Surgery, Queen Elizabeth II Health Sciences Centre and Dalhousie University, Halifax, NS Canada; 2 Department of Pharmacology, Dalhousie University, Halifax, NS Canada; 3 Department of Pathology, Queen Elizabeth II Health Sciences Centre and Dalhousie University, Halifax, NS Canada

**Keywords:** HPV, p16, Oropharyngeal squamous cell carcinoma, Hippo, YAP, Scribble, NOS1AP

## Abstract

**Background:**

HPV infection causes cervical cancer, mediated in part by the degradation of Scribble via the HPV E6 oncoprotein. Recently, Scribble has been shown to be an important regulator of the Hippo signaling cascade. Deregulation of the Hippo pathway induces an abnormal cellular transformation, epithelial to mesenchymal transition, which promotes oncogenic progression. Given the recent rise in oropharyngeal HPV squamous cell carcinoma we sought to determine if Hippo signaling components are implicated in oropharyngeal squamous cell carcinoma.

**Methods:**

Molecular and cellular techniques including immunoprecipiations, Western blotting and immunocytochemistry were used to identify the key Hippo pathway effector Yes-Associated Protein (YAP)﻿1. Oropharyngeal tissue was collected from CO_2_ laser resections, and probed with YAP1 antibody in tumor and pre-malignant regions of HPV positive OPSCC tissue.

**Results:**

This study reveals that the Scribble binding protein Nitric Oxide Synthase 1 Adaptor Protein (NOS1AP) forms a complex with YAP. Further, the NOS1APa and NOS1APc isoforms show differential association with activated and non-activated YAP, and impact cellular proliferation. Consistent with deregulated Hippo signaling in OPSCC HPV tumors, we see a delocalization of Scribble and increased nuclear accumulation of YAP1 in an HPV-positive OPSCC.

**Conclusion:**

Our preliminary data indicates that NOS1AP isoforms differentially associate with YAP1, which, together with our previous findings, predicts that loss of YAP1 enhances cellular transformation. Moreover, YAP1 is highly accumulated in the nucleus of HPV-positive OPSCC, implying that Hippo signaling and possibly NOS1AP expression are de-regulated in OPSCC. Further studies will help determine if NOS1AP isoforms, Scribble and Hippo components will be useful biomarkers in OPSCC tumor biology.

## Background

Oropharyngeal squamous cell carcinoma (OPSCC) has traditionally been a disease associated with long-term use of tobacco and alcohol. In recent decades there has been a shift in the demographics of the OPSCC patient: the majority are now younger, otherwise healthy, non-drinkers and non-smokers. It is widely accepted that this shift is secondary to infection with Human Papilloma Virus (HPV). HPV-16 is commonly associated with a high risk of carcinogenesis, and is found in up to 90% of HPV positive OPSCCs [1–3]. The molecular mechanism of HPV-induced carcinogenesis has been well studied in cervical cancer and there is a growing body of literature concerning its effects in the oropharynx.

HPV is a small virus that infects squamous epithelium. It gives rise to two clusters of proteins: early (E1-7) and late (L1-2). The early genes E5, E6, and E7 all give rise to oncoproteins with the remaining genes coding for regulatory and structural proteins [4]. The oncoprotein E6 causes ubiquitin mediated degradation of the tumor suppressor P53, leading to a reduced rate of apoptosis [4]. The oncoproteins from low-risk HPV strains (e.g. HPV-6) are unable to target tumor suppressor proteins as efficiently as high risk strains such as HPV-16 [4].

In addition to E6 degrading p53, recent studies have shown a direct interaction between the HPV E6 protein and the tumor suppressor protein Scribble, leading to the degradation of Scribble [5, 6]. This interaction initiates epithelial to mesenchymal transition (EMT) transition, an early event in cellular transformation and oncogenesis [7].

Epithelial polarity is a fundamental process in cellular growth and contact inhibition. Disruption of cellular polarity is a major contributor to carcinogenesis. Scribble is a tumour suppressor protein that localizes to the basolateral margins of polarized epithelial cells and plays a major role in establishing cellular polarity [8]. Scribble has also been linked to the intracellular transduction pathway known as Hippo [9, 10]. Activation of the well established Hippo cascade leads to the phosphorylation and inactivation of Yes Associated Protein (YAP)﻿1﻿, ﻿(﻿hereafter referred to as YAP) and its retention in the cytoplasm, whereas YAP dephosphorylation and activation locates it in the nucleus where it drives cellular proliferation [10]. Deregulation of the Hippo pathway occurs in a broad range of human carcinomas, including lung, colorectal, breast, ovarian, pancreatic, gastric and liver cancers [11–19]. YAP deregulation has been implicated in other head and neck malignancies [20, 21], and its expression has been associated with poor patient survival in esophageal cancers [20]. Increased YAP levels and nuclear sequestration were associated with high-grade oral squamous cell carcinoma (OSCC) [21], but it is currently unknown if Hippo pathway deregulation plays a role in OPSCC.

The improved survival and increased susceptibility of HPV positive OPSCC to treatment has led to optimism﻿. Improved understanding of the mechanisms of HPV modulated oncogenesis by different molecular carcinogenic pathways can help in better understanding of tumorgenesis and may lead to more effective targeted therapy in the future. Given the recent rise in oropharyngeal HPV related squamous cell carcinoma and the link between Scribble-NOS1AP and HPV, we sought to determine if Hippo signaling is implicated in such cancers. This study is the first to investigate Hippo signaling and Scribble-NOS1AP disruption in OPSCC.

## Methods

### Samples Selection

P16, HPV positive OPSCC patient tissue was selected randomly from the samples in the Anatomical Pathology Department at the QEII Health Science Center. Any patient less than 18 year old, with HPV status uncertainty or non- oropharyngeal squamous cell carcinoma were excluded from the study. Tissue specimens were acquired from CO_2_ laser wide local resection of OPSCC. Malignant regions, as well as adjacent epithelia, were defined by an onsite histopathologic examination. A portion of the tissues were sectioned and used for hematoxylin and eosin (H&E) staining and immunofluorescence imaging. Sections (5 micron) of tissue were placed on OptiPlus Positive-Charged Barrier Slides.

### Reagents

All chemicals were purchased from Sigma, unless otherwise noted.

### Antibodies

YAP is a mouse monoclonal antibody raised against recombinant human YAP (Santa Cruz, sc-101199); Scribble (H-300) (Santa Cruz – sc-28737) is a rabbit polyclonal antibody raised amino acids 1331-1630 mapping at the C-terminus of human Scribble; pLATS (Ser 909) Cell Signaling (#9157). Pan-NOS1AP and the pep-NOS1APc and GST-NOS1APc antibodies have previously been described [22, 23].

### Cell culture and transfections

Human embryonic kidney HEK293T or MCF7 cells were grown at 37 °C with 5% carbon dioxide in Dulbecco's modified Eagle's medium (DMEM) supplemented with 10% heat-inactivated fetal bovine serum (FBS), 2 mM L-glutamine, 100 U/ml penicillin, and 100 μg/ml streptomycin.

### Immunoprecipitations and Western blotting

Cell lines were homogenized in NP-40 lysis buffer (10% glycerol, 1% NP-40, 20 mM Tris [pH 8.0], 37.5 mM NaCl) containing 1 mM phenylmethylsulfonyl fluoride (PMSF), 10 μg/ml aprotinin, and 10 μg/ml leupeptin. Immunoprecipitation (IP) and Western blotting experiments were performed as previously reported [22]. For analysis of YAP and phospho-YAP levels in ﻿HEK293T cells, protein concentration in whole-cell lysates was quantified using the Bradford assay [24], and lysates were prepared for immunoprecipitation (IP) and Western blotting.

### Immunohistochemistry (IHC)

Tissues were embedded in paraffin then cut in thin sections and preserved for further staining. The sections were then deparaffinized in xylene followed by rehydration through graded ethanol/water until rehydrated in phosphate buffered saline (PBS). Sections were then blocked in 5% normal goat serum containing 0.1% Triton X-100 in PBS. They were then incubated in blocking solution containing anti-YAP and anti-Scribble antibody overnight at 4°C. They were then washed 3 times in PBS followed by incubation with fluorescently labeled secondary antibodies for 1 h at room temperature. Sections were extensively washed in PBS, incubated in Hoechst 33342 (Life Science Technologies), suspended in PBS prior to mounting onto slides and imaging. All images were captured on a Leica DM6100 inverted microscope with appropriate filter sets using Intelligent Imaging Innovations (3i) software for acquisition. All post hoc imaging was done with Photoshop 6.0.

### Thymidine incorporation studies

A thymidine incorporation assay was used to analyze serum-induced proliferation of YFP, YFP-NOS1APa or YFP-NOS1APc stable cell lines as described previously [23]. The incorporation of 1 μCi/ml radioactive [*methyl*-^3^H] thymidine (Perkin-Elmer) into cellular DNA during a 15-min pulse time was quantified by liquid scintillation counting. The entire assay was repeated in two independent experiments, and the data are the averages from four independent replicates in each experiment.

## Results

### NOS1AP and Scribble associate with the Hippo signaling component YAP

**Fig. 1:**
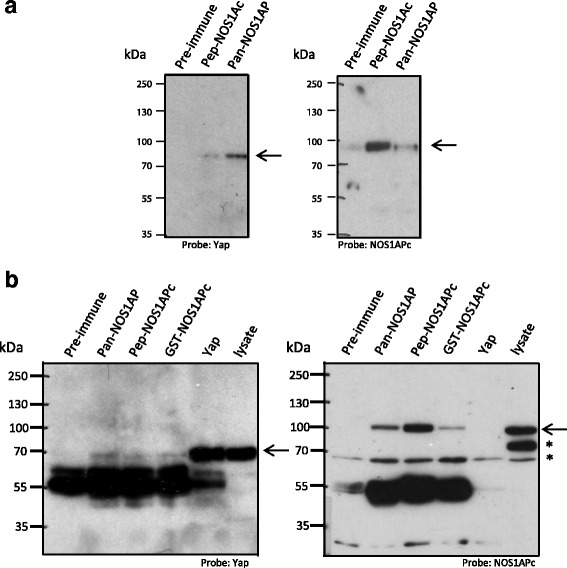
NOS1AP isoforms associate with YAP. **a** HEK293T cell lysate was Immunoprecipitated with the antibodies indicated. The resulting blot was probed with anti-YAP (arrow, left panel) and re-probed with a NOS1APc specific antibody (pep-NOS1APc) (arrow, right panel). Note, more YAP associates with the pan-NOS1AP antibody than with the NOS1APc antibody. **b** Rat brain cell lysate was immunoprecipitated with the indicated antibodies. The resulting blot was probed with anti-YAP antibody (arrow, left panel) and then re-probed with a NOS1APc specific antibody (pep-NOS1APc) (arrow, right panel). Asterisk denote cross reacting bands

The tumor suppressor protein Scribble associates with YAP and regulates Hippo signaling [25, 26]. In a recent report [23], we showed that the nitric oxide synthase 1 adaptor protein (NOS1AP), and an extended splice variant, NOS1APc, associate with Scribble. Further, we have showed that NOS1AP can associate with YAP [23]; however, whether both NOS1APa and NOS1APc associate with YAP and regulates Hippo signaling remains unknown. This is relevant as both NOS1APa and NOS1APc have different subcellular localizations within the cell and associate with different protein complexes [23]. Here we tested whether NOS1APa and NOS1APc could precipitate YAP equally. To test this, we immunoprecipitated equal amounts of lysate from either 293T cells (Fig. 1a) or rat brain lysate (Fig. 1b) with either a pre-immune antibody, a NOS1APc isoform specific antibody (pep-NOS1APc) or a pan-NOS1AP antibody, which detects multiple NOS1AP isoforms, including NOS1APa [23]. The resulting precipitates were then probed for YAP. The pan-NOS1AP and NOS1APc specific antibodies precipitated YAP﻿, ﻿as previously shown ﻿[23]. However, ﻿the NOS1APc antibody precipitated less YAP in both the 293T ﻿cell﻿s and in the rat brain lysates, relative to the pan-NOS1AP antibody (arrow, left panel, Fig. 1a), suggesting that NOS1APc does not associate with YAP to the same extent as some of the other NOS1AP isoforms.

### Distinct NOS1AP isoforms associate with phosphorylated YAP

**Fig. 2:**
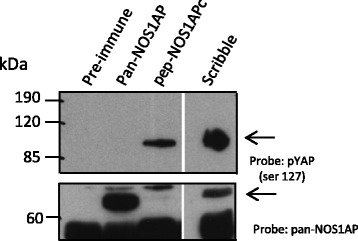
NOS1APc associates with pYAP. Rat brain cell lysate was immunoprecipitated with the antibodies indicated the resulting blot was probed with the antibodies indicated (upper panel pYAP (ser 127). Lower panel, blot was re-probed with a pan-NOS1AP antibody to identify NOS1APa. Asterisks denote ﻿cross reacting﻿ band

Activation of Hippo signaling induces a kinase-signaling cascade leading to the phosphorylation of conserved serine residues in YAP. Since we found that YAP associated differentially with different NOS1AP isoforms, we next wanted to determine if NOS1AP isoforms could preferentially associate with phosphorylated or non-phosphorylated YAP. To test this, we precipitated the different NOS1AP isoforms with the pan-NOS1AP or NOS1APc isoform specific antibodies (pep-NOS1APc) and probed for phosphorylated YAP (pYAP). Interestingly, the pep- NOS1APc antibody was able to precipitate pYAP while the pan-NOS1AP antibody showed little or no recognition for pYAP (arrows, upper panel, Fig. 2). Since Scribble has been shown to interact with YAP [27], we wanted to test whether Scribble could also interact with pYAP. Indeed, Scribble was able to associate with pYAP  (upper panel, Fig. 2). Together this suggests that the different NOS1AP isoforms preferentially associate with either non-phosphorylated or phosphorylated YAP, whereas Scribble can associate with both.

### NOS1AP regulates cellular proliferation

**Fig. 3:**
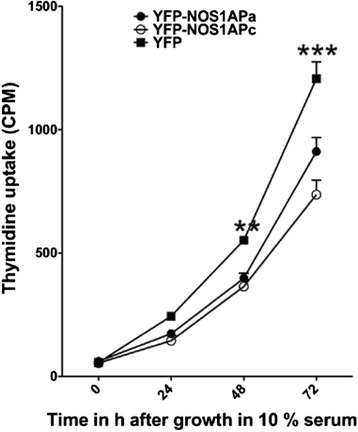
NOS1AP isoforms affects cellular proliferation. Starved MCF7 cells stably expressing YFP-NOS1APa and YFP-NOS1APc incorporate less radiolabeled thymidine over a 48 and 72h period with 10% serum relative to MCF7 cells stably expressing YFP. Differences were considered significant in Students *t*-test at **P < 0.01, ***p < 0.001

Given that Hippo signaling is linked to cellular proliferation and that the differential associations of the NOS1AP isoforms with non-phosphorylated or phosphorylated YAP, we next considered whether overexpression of NOS1AP isoforms would affect cellular proliferation. Here MCF7 cells were used as deregulation of either Scribble or Hippo signaling affects proliferation in these cells. MCF7 cells stably expressing YFP, YFP-NOS1APa or YFP-NOS1APc were monitored for proliferation rates in 10% serum following serum starvation. Interestingly, NOS1APa and NOS1APc showed significantly lower levels of thymidine incorporation compared to YFP at 48 h (**p < 0.01) and at 72 h (***p < 0.001) in 10% serum (Fig. 3). However, no significant difference between YFP-NOS1APa and YFP-NOS1APc was observed at indicated time points (Fig. 3). Together, t﻿his supports the notion that both NOS1AP isoforms affect cellular proliferation and may function as a tumor suppressor.

### YAP accumulates in the nucleus of HPV-OPSCCs

**Fig. 4:**
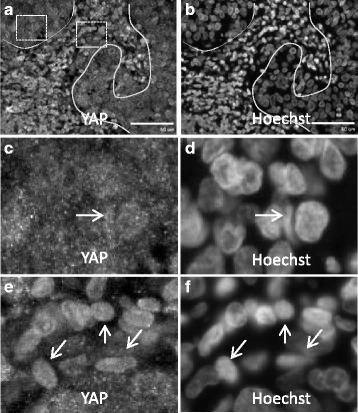
YAP is activated in HPV^+ve^-OPSCC. **a**, **b** HPV^+ve^ -OPSCC stained with anti-YAP (**a**) and Hoechst (**b**). Note: Solid line is tumor margin (**a**, **b**). Boxed regions in (**a**) are expanded in (**c**) left box and (**e**) right box. (**c**, **d**) Enlarged region ( left box in **a**) showing Yap (**c**) and nuclei (**d**). Note, Yap is mainly localized to small puncta in the cytosol in tumor margins, with some cells showing YAP accumulation in the nucleus (**c**, **d** arrow). **e**, **f** Enlarged region (right box in **a**) showing Yap (**e**) and nuclei (**f**). Note, Yap is localized in the nucleus in tumor (**e**, **f**, arrows). Scale bar = 50um

In cervical cancers, HPV infections have been shown to lead to the degradation of Scribble leading to EMT transition [18]. Since Scribble associates with NOS1AP and both Scribble and NOS1AP regulate Hippo signaling, we next tested whether HPV positive OPSCC showed deregulated Hippo signaling. Here sections from an HPV positive OPSCC were stained with YAP (Fig. 4a–f). In malignant cells, the transcriptional co-activator YAP was found in the nucleus (Fig. 4e and f, arrows). Notably, in adjacent pre-cancerous tissue, although most of the YAP was cytosolic, some YAP was observed to accumulate in the nucleus. In non-cancerous tissue, YAP was restricted from the nucleus (Fig. 4c and d).

## Discussion

The Hippo Pathway is a tumor suppressor pathway that was first described in Drosophila. It is involved in coordinating numerous proteins involved in diverse biological processes that have been implicated in terminal differentiation, and deregulation of this pathway leads to cancer [8]. It has been shown in vitro that human Scribble is targeted for degradation by the high-risk HPV E6 proteins [5]. More than 90% of endocervical cancers are HPV related [28]. Nakagawa et al. was the first to describe a link between Scribble and HPV [17]. Subsequent studies have linked higher-grade human endometrial lesions to higher degradation of Scribble protein [29]. Recently, Hippo signaling has been linked to various cancers, and scribble has been shown to be an important mediator of Hippo signaling [25, 26]. In the current study, we sought to test if the human homologue of Scribble is involved in HPV positive OPSCC. We were able to demonstrate that Scribble [23] and its associating protein NOS1AP both precipitate YAP. Further, we found that different NOS1AP isoforms associate with non-phosphorylated YAP and phosphorylated YAP. The exogenous expression of both NOS1APa and NOS1APc affect cellular proliferation suggesting that both NOS1AP isoforms are involved in cellular growth. Finally, our preliminary results suggest a link between the Hippo pathway and the Scribble-NOS1AP axis in HPV related OPSCC. Notably, we found enrichment of YAP in the nucleus of malignant cells in HPV OPSCC, which was absent in benign adjacent cells (Fig. 4) implicating that YAP might provide a useful biomarker in HPV-OPSCC.

In cultured epithelial cells, the formation of cell-cell contacts is an important step in the terminal differentiation before the development of distinct apico-basal surfaces [10, 30]. Recently, the transcriptional co-activator YAP was  implicated in this process [10]. A number of groups have now shown that cell-cell contacts induce the activation of the LATS1/2 serine/threonine kinases that phosphorylate YAP [31–33]. Upon phosphorylation, YAP becomes restricted from the nucleus preventing the transcription of genes important in cell cycle progression [9.10]. A recent cervical cancer study showed that in high-grade cervical lesions nuclear levels of YAP are increased [34]. Additionally, nuclear YAP levels are also increased in premalignant oral lesions [35]. YAP has been found to be an independent prognostic marker for overall survival in liver cancer [36], and Xiao et al., showed that YAP can function as a predictive marker for cervical cancer [34]. These considerations suggest that discerning to what extent Scribble, NOS1AP and the Hippo pathway intersect with HPV-mediated OPSCC will be an important goal for future work.

The HPV E6 protein is known to directly associate with Scribble and induce its degradation through proteasome mediated degradation; however, how this is linked to Hippo signaling activity remains unclear. Recent work has demonstrated that both the tumor suppressor functions of Scribble and the Hippo pathway are genetically linked to tissue growth regulation, and EMT transition [9, 37, 38]. We suggest one important avenue of research is to focus on the adaptor protein NOS1AP. Here we have shown that the NOS1AP isoforms associate with Hippo signaling components as well as with Scribble. Further, we show a differential association of NOS1AP isoforms with cytoplasmic localized and nuclear accumulated YAP. Similarly, we also show that Scribble associates with both differentially localized forms of YAP (Figs. 1 and 2). Whether these interactions with YAP are related to the distinct subcellular localizations of the different NOS1AP isoforms remains to be determined. Interestingly, in neurons endogenous NOS1APa has been reported in the nucleus where non-phosphorylated YAP is found [39]. However, stable expression of NOS1APa is membrane localized in epithelial cell lines, and co-distributes with cytoplasmic YAP [23]. Thus, whether YAP can associate with NOS1APa at cell-cell contacts in a non-phosphorylated or phosphorylated state remains to be explored. Despite the association of the different NOS1AP isoforms with distinct localized YAP pools, both NOS1APa and NOS1APc are capable of reducing cellular proliferation, consistent with previous work showing that NOS1APa functions as a tumor suppressor in breast cancer models [40]. How NOS1AP functions to control Hippo signaling will be an important future focus in cancer biology.

The recent significant rise of HPV related OPSCC has led to a search for alternative pathways that might be more amenable to targeted therapies in future. It is also foreseeable that some of the involved proteins could be subjects for tumor diagnostics and prognosis. To date, we have observed this finding in one patient with HPV positive OPSCC with YAP localizing to the nucleus in tumor tissue. This work follows from another recent study showing that YAP levels are increased in HNSCC [41]. Our current work extends this by demonstrating that in a HPV-OPSCC patient, YAP is localized in the nucleus, suggesting that Hippo signaling is possibly an important signaling pathway with relevance to HPV positive OPSCC, and to HNSCC in general. ﻿Further understanding of the signaling pathwa﻿ys utilized by the different NOS1AP isoforms may provide insight into the underlying biology of OPSCC, and may be an important biomarker in this context﻿. As such, more patients with HPV-positive OPSCC are being recruited to extend our findings and to develop a sense of how the Scribble-NOS1AP-Hippo signaling cascade may differ according to cancer grade.

## Conclusion

In this study the tumor suppressor protein Scribble associates with both YAP and phosphorylated YAP. Interestingly, the Scribble associating protein NOS1AP shows differential association with transcriptionally active and inactive YAP, indicating that distinct isoforms cooperate with the Hippo pathway to inhibit YAP. Further we show that YAP localizes to the nucleus in HPV positive OPSCC while in the pre-malignant tissues YAP is not enriched in the nucleus. Together our study implicates the potential importance of the Hippo pathway in OPSCC and points to the NOS1AP isoforms as potential biomarkers in this process.

## Abbreviations

EMT: Epithelial to mesenchymal transition
H&E: Hematoxylin and eosin
Hoechst 33342: NOS1AP Nitric Oxide Synthase 1 Adaptor Protein
HPV: Human papilloma virus
IHC: Immunohistochemistry
LATS: Large Tumor Suppressor
OPSCC: Oropharyngeal squamous cell carcinoma
OSCC: Oral squamous cell carcinoma
PBS: Phosphate buffer solution
YAP: Yes Associated Protein
